# Walking the Walk: The Case for Internal Equity, Diversity, and Inclusion Work Within the Canadian Public Health Sector

**DOI:** 10.1089/heq.2019.0008

**Published:** 2019-05-02

**Authors:** Ankur Shahi, Fareen Karachiwalla, Nagma Grewal

**Affiliations:** ^1^ Department of Medicine, Queen's University, Kingston, Canada.; ^2^ Department of Family Medicine, Queen's University, Kingston, Canada.; ^3^ Department of Public Health Sciences, Queen's University, Kingston, Canada.

**Keywords:** equity, diversity, inclusivity, public health, workplace

## Abstract

Equity is fundamental to public health practice. However, limited work has evaluated public health units, as employers, in ensuring equitable workplaces. Public health units must examine their policies for promoting equity, diversity, and inclusiveness. We suggest strategies that these organizations may adopt to establish a diverse workforce, including programs of responsibility, broader advertisement of employment opportunities, and standardized application processes. These practices are site dependent and are more effective when supported by senior management. By considering these strategies, institutions of public health can improve equity, diversity, and inclusion in their workplaces while addressing health equity in the communities they serve.

## Introduction

Action on social determinants of health to advance health equity is a fundamental part of public health practice. The Public Health Agency of Canada established the National Collaborating Centre for Determinants of Health (NCCDH) in 2005 to engage in knowledge translation and support health equity work among public health agencies. Recently, Ontario's Ministry of Health and Long-Term Care released new standards for the 35 boards of health. Health equity was named as one of four “foundational standards,” indicating its centrality to public health practice.^1^ This was a welcomed move by many and put equity work at the forefront of public health practice in the province.

The ministry's standards encourage local health agencies to report on inequities, ensure services target priority populations, and build healthy public policy in the communities they serve.^1^ However, there is no explicit mention of the need to assess how health units, as employers, may influence equity. As publicly funded institutions that are accountable to the public, these organizations must evaluate their role in advancing equity in the workplace. A critical need exists for public health agencies to examine their institutional standards and ensure that they “walk the walk” as they move forward with addressing health inequities.

Individuals belonging to underrepresented groups face disparities in employment outcomes in the Canadian labor market. These disparities are based on gender, sexual orientation, race, disability, etc., and are not attributable to skills and qualifications.^2^ It can be difficult to identify discrimination in the labor market, as it is systemic and embedded into organizational culture. However, race, ethnicity, class, and the neighborhood in which one resides are known factors in employers' evaluation of applicants. For example, a study conducted by the Poverty and Employment Precarity in Southern Ontario research group examined the influence of race and a university degree on workers' employment status within the Greater Toronto and Hamilton Area.^3^ This study found that white males and females with a university degree are significantly less likely to report periods of unemployment exceeding 8 weeks when compared with their racialized counter parts with university degrees.^3^ Similarly, a study was conducted to compare wage differences in Canada. After accounting for accumulated human capital, current labor market activity, immigration, language, location, aboriginal status, marital status, self-employment status, and occupational level, the study found that members of visible minority groups earn 14% less than other Canadians.^4^

In addition to the biases mentioned above, institutional practices—such as job advertisement and recruitment practices—can perpetuate a lack of equity, diversity, and inclusiveness. Employers prefer personal social networks and internal candidates over ads or formal sources to recruit in a more timely and cost-effective manner. This hiring practice may pose barriers for individuals without established employment networks from attaining employment.^5^ Lastly, workforce inequities are perpetuated by work environments that do not explicitly embrace equity, inclusiveness, and diversity principles and thus deter applicants from applying.

For public health institutions across Canada to ensure they do not mirror these trends, a few key steps can be taken. First, internal institutional assessments must be conducted. These assessments should explore whether policies exist to address employment equity. The nonprofit HR Council of Canada has proposed questions for such internal evaluations. These questions include (1) What are the characteristics of the community we serve? (2) How has the community changed in recent years? (3) How do our organization's employees reflect the communities we work with? (4) How do we nurture inclusion to ensure all employees work in a safe and supportive environment?^6^ Organizations may also collect employee demographic data and track how these influence job retention, quality, sustainability, and average salary.^7^ It is important to recognize, however, that asking employees to self-identify as members of the nondominant work culture requires a safe and supportive work environment. The results of internal assessments can establish where the public health organization stands and may aid in monitoring progress toward equity, diversity, and inclusiveness.

Several policies exist to help promote employment equity and their effectiveness is site dependent. The potential unintended consequences of such policies are described hereunder and should be given due consideration. These interventions should be incorporated into a comprehensive plan in agreement with staff, rather than as standalone measures. Improving workplace equity, diversity, and inclusion also requires significant commitment from senior leadership and must be adequately resourced.

In 2006, Kalev et al. categorized diversity programs and compared their value in increasing the representation of minority groups. These authors examined three categories of diversity programs: (1) structures establishing responsibility (affirmative action, diversity committees, and diversity staff positions), (2) programs targeting behavioral change through education and feedback (diversity training and diversity evaluation), and (3) programs that improve social networking among minority groups (networking and mentoring programs).^8^ Structures establishing responsibility assign staff to ensure equal representation.^8^ Programs that target behavioral change target unconscious bias.^8^ Programs addressing social networks provide minority groups with opportunities and information required to attain higher level jobs.^8^ Structures that establish responsibility (affirmative action, diversity committees, and diversity staff positions) were found to be the most effective in increasing workplace diversity. To make best use of such programs, organizations should employ appropriate resources and staff.

Alternatively, many organizations have implemented mandatory diversity training to encourage employees to examine their own biases.^9^ The positive impacts of these programs are short lived. The compulsory nature of these courses can also lead to animosity toward equity-seeking groups.^9^ These results highlight that although antidiscrimination training helps to build a foundation, it cannot be a mandatory standalone intervention.

Other strategies that address employment inequities include recruiting more broadly. To encourage applicants from diverse backgrounds, organizations may include a statement of commitment to fostering a diverse environment. While advertising positions, employers should use sources including community boards, employment centers, and organizations that serve ethnic communities, instead of conventional methods. Employers should also implement standardized application systems to ensure that candidates have an equal opportunity to make their interests and qualifications known. Lastly, senior level managers should be held accountable for implementing fair hiring policies, which should be subjected to audits.^2^

A controversial practice that Canadian employers have trialed is resume blinding. A study conducted in 2015 in the United States analyzed outcomes of hiring between two groups (1) in which employers were blind to the identity of the applicant and (2) in which employers were aware of the identity of the applicant.^10^ The identity-blind approach ensured that hiring was conducted based on the individual's achievements without assigning him or her to a demographic group. The identity-conscious group revealed the applicant's identifying data to the employer. Blinding employers to the applicant's identity led to an increase in the employment of women and people of color.^10^ Decreased bias in the identity-blind group may be attributed to employers considering only job-relevant qualifications rather than demographic factors.^10^ However, other sources suggest that underrepresented groups may not be in favor of this strategy, as it delays bias until later in the recruitment process (e.g., interview).^11^ Moreover, it may lead individuals to remove affiliations with groups that indicate membership of a historically underrepresented group (e.g., a cultural organization), thus compromising their self-identity and potentially perpetuating the problem.

The NCCDH is an example of a public health institution that has successfully started the conversation about equitable practices. Connie Clement, the Scientific Director at NCCDH, has written blogs to recognize that there is minimal teaching around racism and health and how internal public health practice may contribute to this cause.^12^ In response, the staff at NCCDH have committed to build an understanding of racism and develop skills to recognize it within their organization.^12^ The staff take part in workshops and informal sessions in which they discuss issues of racism in the field of public health and how to modify their practice to prevent such issues.^12^

Public health units within Canada should use the NCCDH's efforts as an example to increase workplace equity. Examples of practices include multifaith calendars, allowing staff to observe their days of faith; disclosure of preferred pronouns; and explicit statements recognizing the organization's commitment to equity, diversity, and inclusion. There is limited literature examining these individual practices, however, when applied with other initiatives, they can create a welcoming environment for individuals belonging to equity-seeking groups.

## Conclusion

A comprehensive list of interventions to improve employment equity is yet to be developed. However, it is timely to consider effective strategies, as the Canadian Public Health Association (CPHA) recently outlined a set of actions they will take to identify and remove practices that contribute to racism. As a starting point, public health units must conduct internal institutional assessments on equity, diversity, and inclusion in their organizations. Senior organization leaders should engage in discussions regarding long-standing structures and practices that may perpetuate inequities. Lastly, despite deficiencies in the literature, leaders should learn from organizations such as the NCCDH and re-establish the role of Canadian public health agencies in improving equity, diversity, and inclusion in the workplace.

## Abbreviation Used

NCCDH: National Collaborating Centre for Determinants of Health
